# Metabolic scaling: consensus or controversy?

**DOI:** 10.1186/1742-4682-1-13

**Published:** 2004-11-16

**Authors:** Paul S Agutter, Denys N Wheatley

**Affiliations:** 1 Theoretical and Cell Biology Consultancy, 26 Castle Hill, Glossop, Derbyshire, SK13 7RR, UK; 2 BioMedES, Hilton Campus MG7, Aberdeen AB24 4FA, UK

**Keywords:** metabolic rate, allometric scaling, power laws, supply networks, fluid flow

## Abstract

**Background:**

The relationship between body mass (M) and standard metabolic rate (B) among living organisms remains controversial, though it is widely accepted that in many cases B is approximately proportional to the three-quarters power of M.

**Results:**

The biological significance of the straight-line plots obtained over wide ranges of species when B is plotted against log M remains a matter of debate. In this article we review the values ascribed to the gradients of such graphs (typically 0.75, according to the majority view), and we assess various attempts to explain the allometric power-law phenomenon, placing emphasis on the most recent publications.

**Conclusion:**

Although many of the models that have been advanced have significant attractions, none can be accepted without serious reservations, and the possibility that no one model can fit all cases has to be more seriously entertained.

## Introduction: Kleiber and metabolic scaling

In 1932, Kleiber published a paper in an obscure journal [1] showing that standard metabolic rates among mammals varied with the three-quarters power of body mass: the so-called "elephant to mouse curve", termed "Kleiber's law" in this review. Since that date, this and similar allometric scaling phenomena have been widely and often intensively investigated. These investigations have generated continuing debates. At least three broad issues remain contentious, each compounded on the one hand by the problem of obtaining valid data (in particular, finding procedures by which reliable and reproducible measures of standard metabolic rate can be obtained, especially in poikilotherms) and on the other by statistical considerations (in particular, the validity of fitting scattered points to a straight line on a semi-logarithmic plot).

The first issue is disagreement as to whether *any *consistent relationship obtains between standard metabolic rate and body mass. Moreover, those who acknowledge such a relationship hold divergent opinions about its range of application. Is it valid only for limited numbers of taxa, or is it universal? Since the 1960s there has been a measure of consensus: a consistent allometric scaling relationship does exist, at least among homoiotherms. Nevertheless, not all biologists agree, and scepticism is widespread, particularly about the alleged universality of Kleiber's law.

Second, assuming that some version of Kleiber's law (a consistent metabolic scaling relationship) applies to at least some taxa, there are disagreements about the gradient of the semi-log plot. That is, if B = aM^b^, where B = standard metabolic rate, M = body mass, and *a *and *b *are constants, what is the value of *b*? Kleiber [1] and many subsequent investigators claimed that b = 0.75, and on this matter too a measure of consensus has obtained since the 1960s. Once again, however, not all biologists agree. A significant minority of investigators hold that b = 0.67; and other values have been suggested, at least for some organisms.

Third, assuming a consistent scaling relationship and an agreed value of *b*, how is Kleiber's law to be interpreted mechanistically? What is its physical or biological basis? For those who claim that b = 0.67, this issue is simple: standard metabolic rate depends on the organism's surface to volume ratio. But for proponents of the majority view, that b = 0.75, the issue is not simple at all. Many interpretations have been proposed, and since several of these are of recent coinage and seem to be mutually incompatible, a critical comparative review seems timely.

Kleiber's initial paper [1] found support within a decade. The allometric scaling relationship B = aM^b ^(B = standard metabolic rate, M = body mass, *a *and *b *are constants and *b *is taken to be approximately 0.75), was inferred by other investigators during the 1930s [2,3]. Relevant data have been reviewed periodically since then (e.g. [4-15]) and recent developments have rekindled interest in the field.

Many biological variables other than standard metabolic rate also reportedly fit quarter-power scalings (relationships of the kind V = kM^b^, where V is the variable in question, k is a constant and b = n/4; n = 3 for metabolic rate). Examples include lifespans, growth rates, densities of trees in forests, and numbers of species in ecosystems (see e.g. [9]). Some commentators infer that Kleiber's law is, or points to, a universal biological principle, which they have sought to uncover. Others doubt this, not least because it is unclear how (for example) tree densities can be consequences of metabolic scaling or can have the same mechanistic basis. This article focuses on the metabolic rate literature, mentioning other variables only in passing, because most debates in the field have arisen from metabolic rate measurements.

### Variations in the value of *b*

Most debates about the value of *b *assume some version of Kleiber's law: i.e. that a single allometric scaling relationship fits metabolic rates over a wide range of organisms. However, as noted in the introduction, there are dissenters. Everyone acknowledges considerable variation both within and among taxa, no matter whether *b *= 0.75, 0.67, or some other number. The question is whether these variations are deviations from a general law, or whether there is no such law. Conflicting opinions on this fundamental point recall the traditional philosophical difference between physicists and biologists: the former are inclined to see abstract mathematical generalities in any set of numerical data, the latter to see concrete particulars. All recent attempts to explain Kleiber's law by "universal" models have involved physicists and mathematicians; the sceptics are predominantly biologists.

Dodds *et al. *[16] re-examined published scaling data from Kleiber's original paper onwards and concluded that the consensus (*b *= 0.75) was not statistically supported. Feldman [17] found no evidence for any wide-ranging allometric power law in biology and dismissed all attempts to explain scaling relationships by physical or mathematical principles. Atanasov and Dimitrov [18] found evidence that *b *ranges from around 0.67 to more than 0.9 over all major animal groups, the values perhaps reflecting complexity of organisation; single values such as 0.75 emerge only as averages over each group. Other investigators have been less sceptical; publications by Enquist and Niklas [19,20] give particularly impressive support to the generality of Kleiber's law because Niklas was previously among the doubters.

Whatever one's position, it is indisputable that the Kleiber relationship has many exceptions, even among mammals. Bartels [21] showed that some mammals, such as shrews, have B values well above those expected from the Kleiber curve. Andersen [22] discussed the high B values for whales and seals and attributed them to the cold-water habitat. Nevertheless, Kleiber's law has been extended beyond placental mammals to birds and marsupials. Birds have generally higher *a *values than placental mammals and marsupials have lower ones, but the 0.75-power relationship is still inferred by many investigators (e.g. [4]). McNab [13] accepted Kleiber's law as a general *approximation *but emphasized species variations, which he attributed to differences in diet, habitat and physiological adaptation. Elgar and Harvey [23] also found variability among groups of species but reasoned that standard metabolic rates vary taxonomically rather than with temperature regulation, food intake or activity. Economos [24] was also critical of McNab, at least in respect of mammals.

It is difficult to define "standard metabolic rate" in poikilotherms; ambient temperature, time since last meal and other variables markedly affect measurements [9,13,25]. A heterogeneous array of poikilotherm data [5] revealed an "average" *b *value of roughly 0.75. There were wide divergences in some taxa; notwithstanding these, Hemmingsen [4,5] argued that over all animals, plants and protists, metabolic rate scales as the 0.75-power of body mass. More recently published data [26,27] support this conclusion for a wide range of organisms and body masses. However, a careful re-evaluation of Hemmingsen's data by Prothero [28] cast further doubt on the applicability of Kleiber's law to unicellular organisms. Scepticism persists, mostly on the grounds of the intrinsic variability of the data, which is too often underestimated because it is disguised in the customary logarithmic plots and is seldom subjected to adequate statistical analysis [11,29]. However, this too has been debated; a suitable choice of procedures for estimating parameters might eliminate inconsistencies and discrepancies from the data, giving more credence to the belief that b = 0.75 over a wide range of taxa [30]. In the following section we shall examine some of the more divergent data in more detail.

In short, there is a clear but by no means total consensus that (i) Kleiber's law is widely (even universally) applicable in biology, (ii) *b *is approximately 0.75. Variability in the data is generally admitted, so the consensus – and the claim that Kleiber's law manifests a general biological principle – can legitimately be doubted.

### The mass transfer model [31]

Some of the doubts about the consensus are powerfully supported by studies on small aquatic organisms. Reviewing a large literature on metabolic rates in aquatic invertebrates and algae, Patterson [31] deployed chemical engineering principles to explain why the *b *values ranged from about 0.3 to 1.2 in these taxa (his Table 1 provides an excellent summary). Assuming that the delivery of nutrients to each organism entails diffusion through a boundary layer, Patterson showed how water movements and organism size might affect such delivery and hence determine metabolic rate. Using simple geometrical models of organisms (plates, cylinders and spheres), he derived *b *values ranging from 0.31 to 1.25, more or less consistent with the experimental values.

Patterson plotted two dimensionless numbers against each other, *viz*. Sherwood number, Sh = h_m_W/D, where h_m _= mass transfer coefficient, W = characteristic dimension of organism and D = diffusivity; and Reynolds number (a function of organism size), Re = ρUW/μ, where ρ = density, U = water flow speed and μ = coefficient of viscosity. The graphs, which had the form Sh = c.Re^d^, where d = 0.5 for ideal laminar flow and 0.8 for turbulent flow (c is a constant of proportionality), revealed the relative importance of diffusion and mass transfer (convective movement) in the supply of materials. Patterson was able to derive an expression for h_m_, and was thus able to relate the supply of materials to body mass.

The two main attractions of this model are (1) good agreement with a wide range of data and (2) derivation from basic physical principles without *ad hoc *biological or other assumptions. Patterson's approach has implicit support in the literature: Coulson [32] used chemical engineering principles to argue that mammalian metabolic rates are supply-limited, but he did not develop the argument in mathematical detail. However, Patterson's model has drawbacks. First, it is hard to see how his reasoning can be generalised to other taxa, notwithstanding Coulson's proposal (discussed in a later section). Second, by focusing on diffusion and convective mass transfer, he ignored active processes in the uptake of materials, which are likely to dominate in many organisms. Third, he assumed that metabolism in general is supply-limited; in homoiotherms at least, it is more nearly demand-limited under resting conditions, though even this is an oversimplification.

The Patterson model has not been given much attention by other investigators in the field and perhaps it deserves more consideration. Despite its inherent limitations (it is exclusively concerned with small aquatic eukaryotes) it is a potentially fruitful contribution to biophysics.

### Scaling of metabolic rate with surface-to-mass ratio

Several workers accept the reality of allometric scaling but question the value *b *= 0.75, which a consensus of physiologists has accepted since the 1960s. Many of these sceptics claim that the "true" value of *b *is 0.66 or 0.67 because the principal determinant of metabolic scaling is the surface-to-volume ratio of the organism; hence, assuming constant body density, the surface-to-mass ratio. The first study to suggest this explanation for the mass dependence of B is attributed to Rubner [33], who studied metabolic rates in various breeds of dog. Heusner [34] reported that *b *is approximately 0.67 for *any *single mammalian species and suggested that the interspecies value of 0.75 is a statistical artefact. Feldman and McMahon [35] disagreed, but Heusner sustained his position in subsequent articles. For instance, reviewing a substantial body of published data [36], he argued that metabolic rate data for small and large mammals lie on parallel regression lines, each with a gradient of approximately 0.67 but with different intercepts (i.e. values of *a*, termed the "specific mass coefficients"). Hayssen and Lacy [37] found *b *= 0.65 for small mammals and *b *= 0.86 for large ones, again suggesting that *b *= 0.75 is a cross-species "average" with no biological significance; but it is questionable whether their data were measurements of *standard *metabolic rate in all cases. McNab [13] reported lower values: 0.60 and 0.75, respectively. Heusner [36] reasoned that if a few large mammals are added to a sample of predominantly small ones, a single regression line for all the data might have a gradient around 0.75. This, however, is misleading, as the following paragraphs will argue.

According to Heusner, the ratio B/M^0.67 ^is a mass-independent measure of standard metabolism. Variations indicate the effects of factors other than body mass. Other workers broadly share Heusner's opinion (see e.g. [12] for review and [38] for a good recent exemplar). Bartels [21] found a value of 0.66 for mammals; Bennett and Harvey [39] reported 0.67 for birds. Of course, if B varies as M^0.67^, the interesting problem is not the index (*b*) in the Kleiber equation but the allegedly constant relationship between specific mass coefficient (*a*) and body size. This point was developed by Wieser [40], who distinguished the *ontogeny *of metabolism, which comprises several phases but follows the surface rule (M^0.67^) overall, from the *phylogeny *of metabolism, which concerns the mass coefficients (*a*). Following Heusner's argument, Wieser [40] wrote the allometric power law in the form B = a_n_M^0.66 ^and deduced that the specific mass coefficient a_n _= aM^0.09^. Here, *a *is an *interspecific *mass coefficient (3.34 w in mammals if M is in kg). Another difficulty with this type of explanation lies in the calculation of body surface area; the Meeh coefficient, k, where surface area = kM^0.67^, is difficult to measure unequivocally but is generally taken as ~10 (see [3]). Yet another possible difficulty was identified by Butler *et al. *[41], who questioned Heusner's dimensional analysis argument and concluded that no version of Kleiber's law (i.e. no value of *b *that is constant over a range of species) could be substantiated by his approach.

The claim that *b *= 0.67 remains a minority view. Those who accept it are faced with the twin difficulties of (i) establishing that their estimates of surface area are correct and (ii) explaining why, in Wieser's notation, a_n _= aM^0.09^. Moreover, even if such arguments as Heusner's are valid for homoiotherms, it is hard to justify their extrapolation to poikilothermic animals, plants and unicellular organisms, all of which are held by consensus to fit Kleiber's law (but see the two preceding sections). Why should temperature fluxes across the body surface be the main determinants of metabolic rate in poikilotherms, particularly microorganisms? Even in mammals, maintenance of body temperature might not be the main contributor to energy turnover at rest (see later). Contrary to the view of Dodds *et al. *[16], therefore, *b *= 0.67 cannot be treated as a "null hypothesis".

Throughout the remainder of this article, the consensus position will be assumed: Kleiber's law is valid for a wide range of organisms, and b = 0.75. This assumption is made tacitly and provisionally and does not imply dismissal of the foregoing sceptical arguments; but a field can only be reviewed coherently from the consensus point of view.

### McMahon's model [42]

A vertical column displaced by a sufficiently large lateral force buckles elastically. The critical length of column, l_cr_, = k(E/ρ)^1/3^d^2/3^, where d = column diameter, E = Young's modulus and ρ = density. If E and ρ are constant then l_cr_^3 ^= cd^2^, where c is a constant of proportionality. McMahon [42] applied this reasoning to bone dimensions for stationary quadrupeds. In a running quadruped the limbs support bending rather than buckling loads but the vertebral column receives an end thrust that generates a buckling load. It follows that *all *bone proportions change in the same way with animal size. The mass of a limb, w_l_, = αld^2^, where α is a constant. If w_l _is proportional to M, as it generally must be, then M = βld^2^, where β is another constant. Hence (given the above relationship between l and d) M is proportional to l^4^, implying that l is proportional to w_l_^1/4^; hence d is proportional to w_l_^3/8^, or M^3/8^. Empirical support for this relationship appeared in [43].

McMahon [42] also applied this argument to muscles. The work done by a contracting muscle, W, is proportional to σAΔl, where σ is tensile strength, A is the cross-sectional area and Δl is the length change during contraction. The power developed, W/t (t = time), is therefore σAΔl/Δt. Since σ and Δl/Δt are roughly constant and independent of species, W/t varies with A; and since A is proportional to d^2^, W/t it is proportional to d^2^, and therefore to (M^3/8^)^2 ^= M^3/4^. If this deduction applies to any skeletal muscle (as seems plausible), then it applies to the entire set of metabolic variables supplying the muscular system with nutrients and oxygen. Hence, B varies as M^3/4^. A broadly comparable but simpler argument was advanced by Nevill [44]; large mammals have proportionately more muscle mass than smaller ones. If the contribution of the muscle to B (which Nevill assumes is proportional to M) is partialled out, then the residual B is proportional to M^2/3^. Nevill's paper is seldom cited.

One difficulty with McMahon's model is that little of the energy turnover under conditions of standard metabolic rate measurement entails muscle contraction. The model might still be valid if maximum metabolic rate followed the same allometric scaling law as B; this has been widely believed, and Taylor *et al. *[45] adduced evidence for it. However, recent detailed studies [46-48] indicate that maximum metabolic rate in birds and mammals scales as M^0.88^, not M^0.75^, although there are disagreements about whether aerobic capacity determines the allometry of maximum metabolic rate [48,49]. Weibel [50] presented a large set of data to this effect. (On the other hand, there are reports that in birds the index *decreases *rather than increases with increasing metabolic output, e.g. [58].) Another drawback of the McMahon model is that it cannot apply to organisms without muscles, such as protists. This perhaps explains why McMahon's elegant deduction has been largely ignored in recent debates about Kleiber's law.

### The Economos model [51]

An increased gravitational field increases energy metabolism in animals [52,53]. Work against gravity is proportional to M^1.0^. If maintenance metabolism were related to surface area (proportional to M^0.67^) then a combination of the two effects, surface-to-mass ratio and work against gravity, might explain the observed M^3/4 ^relationship. This model [51] is difficult to assess: it is not clear why the two proposed factors, surface area dependence and gravitational loading, should combine for *all *animals (and other taxa) in just the right proportions to generate a 0.75-power dependence on body mass. To take just one example, aquatic microbes are more affected by Brownian motion than by gravity, so why should they show the same balance between surface-to-mass ratio and gravitational effects as mice or elephants? Pace *et al. *[54] suggested that the Economos model could be critically tested under conditions of weightlessness in space. No corroboration (or refutation) by studies on astronauts has been reported.

### Allometric scaling in cells and tissues

Before more recent models purporting to explain Kleiber's law are discussed, some comments are needed on scaling of metabolism at the organ, tissue and cell levels. Belief that the Kleiber relationship can be explained in terms of the inherent properties of the cells dates from the 1930s [3,55] and persists (e.g. [56,57].

Standard metabolic rate (B) is usually measured as oxygen consumption rate, which correlates with nutrient utilization [9,15] and rates of excretion of nitrogenous and other wastes [2]; so research in the field has been dominated by respiratory studies. Lung volume, trachaeal volume, vital capacity and tidal volume all scale as M but respiratory frequency varies as M^-0.31^, ventilation rate as M^0.77 ^and oxygen consumption rate as M^0.72 ^[58-60]. All mammals extract a similar percentage of oxygen (~3%) from respired air [9]. The significance of "pulmonary diffusion capacity" has been debated; it scales as M^1.0 ^so it is disproportionate in bigger animals [17,61-65].

Stahl [60] described the scaling of cardiovascular and haematological data. Blood haemoglobin concentration is the same for all mammals except those adapted to high altitudes. Blood volume is ~6–7% of body volume for all mammals except aquatic ones. Erythrocyte volume varies with species but bears no obvious relationship to M. The oxygen affinity of haemoglobin varies with body size, being lower in smaller mammals, which unload oxygen to their tissues more rapidly. Capillary density is more or less constant in mammals with bodies larger than a rat's, though it is greater in the smallest mammals [65]. The heart accounts for ~0.6% of body mass in all mammals [66]. Heart rate scales as M^-0.25^, cardiac output as M^0.81 ^(60) and circulation time as M^0.25^. The energy cost of supplying the body with 1 ml of oxygen is similar for all mammals [15].

Standard metabolic rate has two main components: service functions, e.g. the operation of heart and lungs; and cellular maintenance functions, e.g. protein and nucleic acid turnover (e.g. [67]). Krebs [68] elucidated this second component by studying tissue slices; his investigation has since been extended. Oxygen consumption per kg decreases with increasing M in all tissues, but tissues do not all scale identically. Horse brain and kidney have half the oxygen consumption rates of mouse brain and kidney but the difference between these species in respect of liver, lung and spleen is 4-fold [68-70]. Metabolic rate in liver scales as M^0.63^; for some organs the exponent is closer to 1.0; the sum of oxygen consumption rates over all tissues gives – approximately – the expected 0.75 index [71]. The difficulty of recalculating B from tissue-slice data is considerable, so the Martin and Fuhrman calculation [71] has wide confidence limits. Spaargen [72] suggested that tissues that use little oxygen constitute different percentages of body mass in large and small mammals, leading to a distortion of the surface law (B = M^2/3^), which would otherwise be valid. More recently, however, Wang *et al. *[73] repeated the Martin and Fuhrman calculation using improved data, and found impressive support for the consensus B = M^3/4^.

Cells of any one histological type are size-invariant among mammals but allometric scaling is reported at the cellular level; e.g. the metabolic rate of isolated hepatocytes scales as M^-0.18 ^[74]. Numbers of mitochondria per gram of liver (or per hepatocyte), however, scale as M^-0.1 ^[75,76]. The apparent discrepancy between these values might be illusory (*cf. *[77]), or it might indicate a greater proton leak in mitochondria from livers of smaller animals [78] or allometry in redox slip [79]. Also, larger animals have smaller inner mitochondrial membrane surface areas (the scaling is M^-0.1^) and different fatty acid compositions [71]. The discrepancy between the scalings of hepatocyte and whole-body metabolism is probably explained by the decrease in liver mass, which scales as M^0.82 ^[75,80]. Combining liver mass with hepatocyte oxygen consumption, the derived scaling for liver metabolism is M^0.82^.M^-0.18 ^= M^0.64^, consistent with the experimental tissue-slice data (M^0.63^; see above). Combining liver mass with mitochondrial number per hepatocyte gives a similar value [77]. Cytochrome c and cytochrome oxidase contents scale roughly as M^0.75 ^[81-85]. The allometric scaling of mitochondrial inner membrane area, and the body-size-related differences in unsaturated fatty acid content, remain unexplained.

Isolated mammalian cells reportedly attain the same mitochondrial numbers and activities after several generations in culture, irrespective of the tissue of origin or the organism's body mass [86-88]. If allometric scaling is lost at the cellular level after several generations *in vitro*, then presumably mitochondrial densities, inner membrane areas and cytochrome levels somehow become "normalized". This is a readily testable prediction [see [89]], but it does not appear to have been subjected to critical experiments. If it is corroborated there will be interesting mechanisms to investigate.

The main conclusions from this section are: (a) different organs make different contributions to the scaling of whole-organism metabolic rates; (b) differences at the cellular level make relatively small contributions to scaling at the organ level; (c) these differences at cellular level might disappear altogether after several generations in culture. The most striking conclusion is (b). It implies that allometric scaling of metabolic rate does not after all, for the most part, reside in cellular function but at higher levels of physiological organisation. If this is the case, then the alleged applicability of Kleiber's law to unicellular organisms is called into question.

### Resource-flow models

Coulson's flow model [42] was mentioned earlier. It relates tissue or organ oxygen consumption rates to circulation times, i.e. to the rate of supply of oxygen and nutrients, and these scale as M^0.25 ^(see previous section). Coulson's approach contrasts with traditional biochemical measurements: the principal variable is not the concentration of a resource but the *supply rate*; metabolic activity depends on *encounter frequency *not *concentration*. This perspective merits further development, particularly by extension to the cell internum [89-93]. Obviously, it is within the cell that the reactant molecules are passed over the catalysts; and the flow rate increases with the cell's metabolic activity, as Hochachka [93] cogently described.

However, flow theories advanced to explain Kleiber's law have not followed this line of argument. Banavar *et al. *[94,95] and Dreyer and co-workers [27,96] have shown that the Kleiber relationship can be deduced from the geometries of transport networks, without reference to fluid dynamics. Broadly, these authors argue that as a supply network with local connectivity branches from a single source (in a mammalian circulatory system, the heart is the source), the number of sites supplied by the network increases. Natural selection has optimized the efficiency of supply. A general relationship can be derived between body size and flow rate in the network: delivery rates per unit mass of tissue vary with the quarter-power of body size (M), implying the validity of Kleiber's law.

The most detailed account of this argument [95] begins with the reasonable assumption that M scales with L^D^, where L is the physical length of the organism and D is its dimensionality. It proceeds with a theorem: the sum of flows through all parts of the network, F, is proportional to the (dimensionless) length multiplied by the metabolic rate. A quantity measuring the total flow of metabolites per unit mass of organism is then defined: r_1 _= F/M. r_1 _(which has units of inverse time) measures the dependence of the network's geometry on body mass, so it indicates the energy cost of metabolite delivery. Another parameter, r_2_, measures the metabolite *demand *by the tissues: r_2 _= the dimensionless length of the "service volume" (the amount of tissue that consumes one unit of metabolite per unit time). It is then deduced that B is proportional to (Mr_1_/r_2_)^D/(D+1)^. Provided that r_1 _and r_2 _change proportionately – i.e. supply always matches demand – then for a three-dimensional organism, Kleiber's law follows. According to Banavar *et al. *[94], deviations from Kleiber's law indicate inefficiency or some physiological compensation process.

This model has been criticized [97] because the assumed network does not resemble (e.g.) the mammalian circulatory system, where only *terminal *nodes (capillaries), not *all *nodes (as the model implies), are metabolite exchange sites. Also, the model seems to predict that r_1_/r_2 _will decrease as B rises from standard to maximal; but the best data suggest the opposite trend (see earlier discussion: [46-48]). Banavar *et al. *do not explicitly allow for differences among tissue types, which are considerable (see above), except perhaps in terms of rather implausible variations among r_1_/r_2 _ratios. On the other hand, the model is simple and flexible and it reflects recent developments in the physics of networks. If it could be applied to flow at the cellular level, it might accord with the requirements discussed at the beginning of this section; though it is difficult to see how this can be achieved.

Rau [98] also advanced a fluid-flow model, but his conception is physical not geometrical. Assuming Pouseille flow through an array of similar tubes, such as capillaries, and a roughly constant flow speed, Rau used scaling arguments to derive the relationship t = kM^1/4^, where t is the transport time and k is a constant. If the fluid transport rate (essentially the reciprocal of t) is proportional to B/M, Kleiber's law follows. However, Rau's model appears to assume that because metabolic rate is energy per unit time, it can be equated with the product of fluid volume flow rate and pressure (since energy is equal to pressure times volume). This assumption, which appears to be based exclusively on dimensional analysis, is fallacious.

### Four-dimensional models

Blum [99] observed that the "volume" of an n-dimensional sphere of radius *r *is V = π^n/2^r^n^/Γ(n/2 + 1), and that A = dV/dr = nπ^n/2^r^n-1^/Γ(n/2 + 1). Here, Γ(n) is the gamma-function such that Γ(n + 1) = n_n_, Γ(2) = 1 and Γ(3/2) = π^1/2^/2. Suppose two objects have "volumes" V_1 _and V_2 _and "areas" A_1 _and A_2_. From the foregoing, A_1_/A_2 _= (V_1_/V_2_)^(n-1)/n^; so if n = 4, a 3/4-power relationship between "volumes" (hence, masses?) emerges from a familiar mathematical principle. Might Kleiber's law therefore follow from a four-dimensional description of organisms? Speakman [100] pointed out that if n = 4, then A is volume (it has three dimensions) and V is hypervolume, the biological significance of which is obscure. However, West *et al. *[88,101,102] have indeed proposed a four-dimensional model to explain the Kleiber relationship, and considerable claims have been made for their account.

This model addresses the supply of materials (particularly oxygen) through space-filling fractal networks of branching tubes. It assumes that as a result of natural selection, organisms maximize their use of resources. The initial account [101] assumed that energy dissipation is minimised at all branch-points in the network and that the terminal branches are size-invariant (for instance, blood capillaries are the same lengths and diameters in mice and elephants). Kleiber's law and analogous scalings were deduced from these assumptions. In particular, the three-quarters-power exponent was shown to be inherent in the geometry of a branching network that preserves total cross-sectional area at each branch point. The circulatory systems of large animals such as mammals are not exactly area-preserving, but West *et al. *[101] reasoned that this objection could be circumvented by considering the pulsatile flow generated in the larger arteries by the action of the heart.

A second, simpler account [102] developed the model from a geometrical basis. The crucial feature of the branching network is the size-invariance of the terminal units. The effective exchange area, **a**, is a function of the element lengths at each level of the hierarchy, but one of these, the terminal one (l_0_), is invariant. Writing Φ as a dimensionless function of the (dimensionless) ratio l_1_/l_2 _leads to

**a **(l_0_, l_1_, l_2_,...) = l_1_^2^Φ(l_0_/l_1_, l_2_/l_1_...)

Introducing a scaling factor, λ, leads to

**a **(l_0_, l_1_, l_2_,...) = λ^2^l_1_^2 ^Φ(l_0_/λ l_1_, l_2_/l_1_...)

which is not proportional to λ^2 ^because l_0 _is fixed. The dependence of Φ on λ is not known *a priori*, but it can be parameterized as Φ(l_0_/λ l_1_, l_2_/l_1_...) = λ^ε^Φ(l_0_/l_1_, l_2_/l_1_...), where ε is between 0 and 1. This power law reflects the fractal character of the network's hierarchical organization. Similar reasoning is applied to body volume, hence body mass, and the following expression for the exchange surface area is derived:-

**a **= kM^r^, r = (2 + ε)/(3 + ε + ζ),

where k is a constant and ζ (0 < ζ < 1) is an arbitrary exponent of length, just as ε is an arbitrary exponent of area. If natural selection has acted to maximize the scaling of **a**, then ε must tend to 1 and ζ to 0. This gives r = 0.75. If **a **limits the supply of oxygen and nutrients, and hence determines standard metabolic rate, then B is proportional to **a **and Kleiber's law follows.

The model has several attractions: it derives from well-established physical principles, invokes natural selection and is mathematically impeccable. It implies that cells and organelles transport materials internally along space-filling fractal networks rather than by "diffusion", which seems correct [83,85,86,103]. The self-similarity of these transport networks is emphasized particularly in [88]. The dimensionalities of effective exchange surfaces, **a**, are predicted to be closer to 3 than 2; empirically, the microscopic convolutions of surfaces such as the mammalian intestinal mucosa are well known. The mass of the smallest possible mammal is deduced and shown to be close to the mass of the shrew. Other approaches to exchange networks, assuming minimum energy expenditure and scale-invariance, have led to similar models [104]. The model can be adapted, with no loss of rigour, to new data: Gillooly *et al*. [105] showed that the fractal supply network principle can be combined with simple Boltzmann kinetics to explain the effects of both body mass and temperature on metabolic rates. Since mass and temperature are the primary determinants of many physiological and ecological parameters, this work suggests that the model [88] could revolutionize biology.

This is an impressive range of successes. However, West and his co-workers make claims that are less compelling. The observation that cytochrome oxidase catalytic rates fit the same allometric curve as whole-organism metabolic rates is claimed as corroboration. However, cytochrome oxidase is not an organism, or a cell: it does not have a metabolic rate. It is also debatable whether mitochondria can be said to have "metabolic rates". (In contrast, Hochachka and Somero [106] noted that oxygen turnover in the whole biosphere can be fitted to the same curve; but they recognized this as "a contingent fact with no biological significance".) Also, the explanation derived by West and his colleagues for the alleged body-mass-invariance of the metabolic rates of cultured cells (see earlier) is mathematically neat, but it leads to no experimentally testable predictions, and the heterogeneous data sources cited in this context make the *explicandum *itself unconvincing. Finally, the model is said to explain the quarter-power scalings of a wide range of biological variables other than metabolic rate, including population densities of trees [19] and carnivorous animals [107], plant growth rates, vascular network structure and maturation times [18,108], and life-spans [88]. It is not clear why any of these variables should depend on the fractal geometries of space-filling supply networks, still less on metabolic rates; though there is widespread interest in the application of scaling laws in ecology, for instance in modelling biodiversity [109] and food webs [110].

Moreover, there are definite flaws in the model:-

(1) If West *et al. *were correct, maximal and standard metabolic rates should both scale as M^0.75^. The weight of evidence suggests that maximum rate in homoiotherms scales as M^0.88 ^(see earlier discussion [46-49] and following section).

(2) During maximal energy output by an organism, the supply of material is likely to be limiting. For example, in mammals, muscle contraction is responsible for most of the energy turnover at maximum output and it is generally believed that the rate is limited by oxygen supply (if anaerobic capacity is ignored). However, under standard metabolic rate conditions, energy *demand *is generally more significant, i.e. for the service and cellular maintenance functions mentioned previously. Therefore, it is not clear why the geometry and physics of the *supply *system should predict the allometric scaling of standard rather than maximal metabolic rate. ("Supply" and "demand" under conditions of maximal aerobic metabolism are complex terms because many physiological steps are involved. The extent to which each step limits the maximum metabolic rate might be quantifiable by a suitable extension of metabolic control analysis [111]; this remains an active research area to which West *et al. *scarcely refer.)

(3) The mathematical derivations given in [101] are idealisations, but they do not seem to allow for large deviations from *b *= 0.75. However, there are often wide differences among empirical *b *values, as discussed earlier; these were addressed in, for example, [18] and [31]. Also, the model does not account, or allow, for the differences in allometric scaling among mammalian tissues and organs [66,73,80].

(4) West *et al. *accept that some of their proposed hierarchical supply networks might be "virtual" (as in mitochondria) rather than explicit (as in mammalian blood circulation), but it is not clear why such networks must always have the same geometry. For instance, why should the intracellular network discussed by Hochachka [93] show area-preserving branching? There is no evidence that it does. Moreover, the "flow" of reductants through mitochondria presumably takes place in the plane of the inner membrane, which has one dimension fewer than (say) the mammalian circulatory system, so even if mitochondria can be said to have "metabolic rates", the 0.75-power law cannot apply here; yet, allegedly, it does apply.

These difficulties show that the West *et al. *model, despite its impressive economy, elegance, consistency and range, cannot be accepted unreservedly in its present form. The very generality, or "universality", of this model has made it suspect for some biologists [25]. The implication that it reveals a long-suspected universal biological principle implicit in Kleiber's law has ensured its attraction for others [14].

### The model of Darveau and co-workers [112]

This group elaborated a multi-cause rather than a single-cause account of allometric scaling. Their "allometric cascade" model holds that each step in the physiological and biochemical pathways involved in ATP biosynthesis and utilization has its own scaling behaviour and makes its own contribution (defined by a control coefficient between 0 and 1) to the whole-organism metabolic rate. Thus, many linked steps rather than a single overarching principle account for Kleiber's law.

This idea is inherently plausible, and the model is attractive because it draws upon recent advances in metabolic control analysis in biochemistry [111] and physiology [113]. It emphasises that standard metabolic rate is determined by energy demand, not supply; and it predicts an exponent for maximal metabolic rate in mammals between 0.8 and 0.9, rather than 0.75, which agrees with experimental findings [46-49] and the data cited by Weibel [50]. Implicitly – though the authors do not emphasize this – it seems capable of explaining *b *values that are far from 0.75 (*cf *[31]). It is hardly surprising, therefore, that many responses to the Darveau *et al. *model have been positive [e.g. [114]].

However, Darveau *et al. *made no attempt to explain *why *the values of b are typically around 0.75, as West *et al. *and others have done. The model is phenomenological, not physical and mathematical; their equations are not derived from any fundamental principle(s). Moreover, their data cover only some three orders of magnitude of body mass, whereas many studies have involved much wider ranges. This might make their overall *b *values misleading [103] or, alternatively, more credible [18]. When their equations are applied to a mass range of eight orders of magnitude, different *b *values are obtained, not necessarily consistent with published data; but on the other hand, the published data might not be correct.

In the first published account of this model [112] the mathematical argument was flawed. The basic equation was given in the form B = aΣc_i_M^b(i)^, where *a *is a constant coefficient, c_I _is the control coefficient of the i^th ^step in the cascade and b(i) is the exponent of the i^th ^step. By definition, the sum of all the c_I _values is unity. Darveau *et al. *did not derive this equation; they stated it. They also stated that the overall exponent, the *b *term in the Kleiber equation, is a weighted average of all the individual b(i) values, the weighting being determined by the relevant control coefficients. It has been suggested that this leads to untenable inferences. For example, since the units of B and *a *are fixed, the units of c_I _must depend on those of b(i); but by definition, both b(i) and c_I _must be dimensionless. Also, according to the basic equation, the contribution made by each step to the overall metabolic rate depends on the units in which body mass is measured. If this criticism is valid then it is impossible to evaluate the model as it stands, because any attempt to align its predictions with experimental data would be meaningless. Another reservation about this model is that it does not purport to apply to all taxa, as the West *et al. *model does; it relates only to metazoa, and in particular to homoiotherms. However, most of the relevant data in the literature concern homoiotherms.

A subsequent publication from this group [115] re-stated the basic equation in the form B = aΣc_I_(M/m)^b(i)^, where the constant *a *is described as the "characteristic metabolic rate" of an animal with characteristic body mass *m*. This eliminates the problem of mass units, because the mass term has been rendered dimensionless; and it is mathematically simple to express control coefficients in dimensionless form. The revised equation might therefore be immune to some of the criticisms levelled at its predecessor. However, some of the earlier reservations remain: the equation remains phenomenological, not physical or geometrical; and the restriction in its range of application is explicit. Nevertheless, these considerations by no means invalidate the model. Indeed, it is supported by data from experiments in exercise physiology [116].

The models of Darveau *et al*. [112,115], Banavar *et al. *[94,95] and West *et al. *[88,102] all have attractive features; but they all have flaws, and they cannot be reconciled with one another. If the positive contributions to biology that these models represent could be further developed, and their defects eliminated, could they be harmonized? If so, the advancement of our understanding would be considerable.

## Conclusions

Several explanatory or quasi-explanatory models have been proposed for the allometric scaling of metabolic rate with body mass. Most of them have significant attractions, particularly the most recent ones, but none of them can be unreservedly accepted. The variability of experimental data leaves room for doubt that Kleiber's law is universally or even widely applicable in biology [17,117], yet most workers in the field presume that it is. Even if such doubts are set aside, no model has yet addressed every relevant issue. For example, the biochemical reasons for the allometric scalings of mitochondrial inner membrane areas and unsaturated fatty acid contents, and the direct proportionality of "pulmonary diffusion capacity" to body mass, remain unexplained. Despite the continuing controversy in the field, the consensus remains, and practical use has been made of Kleiber's law, for example in making numerical predictions of anatomical and physiological parameters for veterinary applications [118]. Perhaps the last word should be given to Bokma [119], whose most recent paper explores the power-scaling of metabolic rate to body mass (*b*) on an intra-specific basis from a total of 113 species. He came to the conclusion that there was no single universal value of *b*. This evidence alone must make us more sceptical of there being some unifying law involved that demands that *b *holds close to 0.75. There is clearly no consensus otherwise *Nature*, *Science *and the *Proceedings of the National Academy of Sciences USA *would cease to publish so regularly many of the articles to which we have referred. The subject is not only unresolved, but remains very much within the general interest of biologists.

Kleiber's law remains a fascinating mystery; possibly a delusion, possibly a widespread or even ubiquitous biological phenomenon for which no entirely satisfactory account has yet been offered. Recent developments, though mutually conflicting as they stand, have the potential to lead to new insights and to uncover one or more general biological principles that will have a profound impact on our understanding of the living world.
